# Novel Carbon Quantum Dots/Silver Blended Polysulfone Membrane with Improved Properties and Enhanced Performance in Tartrazine Dye Removal

**DOI:** 10.3390/membranes10080175

**Published:** 2020-08-03

**Authors:** Jin Yee Gan, Woon Chan Chong, Lan Ching Sim, Chai Hoon Koo, Yean Ling Pang, Ebrahim Mahmoudi, Abdul Wahab Mohammad

**Affiliations:** 1 Department of Chemical Engineering, Lee Kong Chian Faculty of Engineering and Science, Universiti Tunku Abdul Rahman, Jalan Sungai Long, Bandar Sungai Long, Cheras, Kajang 43000, Selangor, Malaysia; cannygan97@1utar.my (J.Y.G.); simcl@utar.edu.my (L.C.S.); pangyl@utar.edu.my (Y.L.P.); 2 Department of Civil Engineering, Lee Kong Chian Faculty of Engineering and Science, Universiti Tunku Abdul Rahman, Jalan Sungai Long, Bandar Sungai Long, Cheras, Kajang 43000, Selangor, Malaysia; kooch@utar.edu.my; 3 Chemical Engineering Programme, Faculty of Engineering and Built Environment, Universiti Kebangsaan Malaysia, Bangi 43600, Selangor, Malaysia; ebi.dream@gmail.com (E.M.); wahabm@eng.ukm.my (A.W.M.); 4 Research Center for Sustainable Process Technology (CESPRO), Faculty of Engineering and Built Environment, Universiti Kebangsaan Malaysia, Bangi 43600, Selangor, Malaysia

**Keywords:** carbon quantum dots, ultrafiltration, hydrophilicity, rejection, anti-fouling

## Abstract

This study produced a novel polysulfone (PSF) membrane for dye removal using lemon-derived carbon quantum dots-grafted silver nanoparticles (Ag/CQDs) as membrane nanofiller. The preparation of CQDs was completed by undergoing hydrothermal treatment to carbonize the pulp-free lemon juice into CQD solution. The CQD solution was then coupled with Ag nanoparticles to form Ag/CQDs nanohybrid. The synthesized powders were characterized in terms of morphologies, functional groups and surface charges. A set of membranes was fabricated with different loadings of Ag/CQDs powder using the nonsolvent-induced phase separation (NIPS) method. The modified membranes were studied in terms of morphology, elemental composition, hydrophilicity and pore size. In addition, pure water flux, rejection test and fouling analysis of the membranes were evaluated using tartrazine dye. From the results, 0.5 wt % of Ag/CQD was identified as the optimum loading to be incorporated with the pristine PSF membrane. The modified membrane exhibited an excellent pure water permeability and dye rejection with improvements of 169% and 92%, respectively. In addition, the composite membrane also experienced lower flux decline, higher reversible fouling and lower irreversible fouling. This study has proven that the addition of CQD additives into membrane greatly improves the polymeric membrane’s properties and filtration performance.

## 1. Introduction

Water is one of the most essential sources of life other than oxygen, food and shelter. It is indispensable to human survival and sustainable development. Hence, the provision of clean water must not be neglected in terms of quantity and quality. The establishment of the sixth sustainable development goal (SDG) is to ensure higher availability and sustainable management of clean water and sanitation for every single person [1]. To achieve this, developing a highly efficient and versatile water treatment processes to remove pollutants is essential. 

The textile industry is one of the major contributors to severe water pollution [2]. Due to the overpopulation rate of human beings, textile products such as garments, curtains, and blankets are highly demanded [3]. Tartrazine, as known as Acid Yellow 23, is an anionic azo dye which is commonly used in textile and paper industries [4]. It is evident that tartrazine is one of the root causes of asthma, urticaria and angioedema [2,5]. To reduce water pollution issues, every industry player should take responsibility in treating the dye waste before they are discharged into the water bodies. Polysulfone (PSF) polymeric membrane is commonly used to produce ultrafiltration (UF) membrane for water and wastewater treatment. PSF polymer has high chemical stability, desirable mechanical and thermal properties, high resistance in a wide range of pH, as well as being highly soluble in a wide range of polar solvents [6,7]. As the polymeric membrane is hydrophobic, modification is required to improve the permeability, rejection and fouling resistance of the membrane [8,9]. 

Carbon quantum dot (CQD) is a novel, zero-dimensional carbon nanoparticle with a size of 10 nm or less, with an abundance of functional groups such as carbonyl, carboxyl and hydroxyl [10]. CQDs are highly water soluble, have high biocompatibility and exhibit excellent optical properties. CQD could be easily produced and its properties could be modified with various functional groups by tuning the precursors and reaction parameters for different applications [11]. Previous studies showed that CQD had considerable promise in a variety of research areas such as biosensing, bioimaging and photocatalytic applications [12]. 

Recently, CQD has been applied in thin film composite (TFC) to enhance the membrane’s permeability, rejection and anti-fouling properties. Gai et al. [13] first explored the possibility of incorporating sodium functionalized CQD in pressure-retarded osmosis membrane for power generation. The novel TFC membrane showed the highest ever power density of 34.20 W/m^2^ compared to previous literature. He et al. [14] synthesized CQD from citric acid and functionalized with sodium to produce TFC via interfacial polymerization. The modified TFC exhibited excellent permeability and superior rejections of over 97% towards arsenic and selenium. Sun and Wu [15] produced carboxylic CQD (CCQD), amino CQD (NCQD) and sulfonated CQD (SCQD) for TFC fabrication and compared their effect on permeability, sodium sulfate (Na_2_SO_4_) rejection and anti-fouling study. It was reported that SCQD-TFC exhibited the highest permeability and the best anti-fouling performance while NCQD-TFC demonstrated better rejection for divalent cations. The excellent hydrophilicity, large surface area, functional groups and favorable polymer affinity of the CQD have contributed to the successful enhancement of the TFC performance. 

The above studies delivered a valuable insight into the application of CQD in membrane fabrication technology. To date, there are a limited number of TFC and UF membranes incorporated with CQD [16,17]. With that, we proposed the embedment of silver/carbon quantum dot (Ag/CQD) nanocomposite for the fabrication of a novel PSF membrane. The natural product-derived CQD from lemon juice was first applied in membrane fabrication. It was impregnated with Ag nanoparticles and their physical and chemical characteristics were studied. Ag nanoparticle is known to have antimicrobial properties, hydrophilicity and negative surface charge. The incorporation of CQD on Ag nanoparticle to further improve its negative surface charge and hydrophilicity was the focus of this study. The impact of the nanocomposite on PSF polymeric was investigated in terms of membrane morphology, hydrophilicity, permeability, rejection and fouling studies. Tartazine dye was applied as the pollutant model to evaluate the rejection and fouling resistance of the membranes in this study.

## 2. Materials and Methods 

### 2.1. Materials

Eureka lemon, ethanol (C_2_H_5_OH, Sigma Aldrich, Darmstadt, Germany), and dichloromethane (CH_2_Cl_2_, Merck, Darmstadt, Germany) were used to synthesize CQD solution. Silver nitrate (AgNO_3_, Sigma Aldrich) and sodium borohydride (NaBH_4_, Sigma Aldrich) were utilized for the synthesis of pure Ag and Ag/CQD nanomaterial. Tartrazine (Dye content ≥ 85%, Sigma Aldrich) was used as the dye pollutant. Besides, sodium hypochlorite (NaOCl, Sigma Aldrich) was applied as membrane cleaning agent. Polysulfone (PSF, Solvay, GA, USA), polyvinylpyrrolidone (PVP, 160,000 g/mol, Sigma Aldrich) and N-methyl-2-pyrrolidone (NMP, Merck) were used for the fabrication of composite membrane.

### 2.2. Preparation of Carbon Quantum Dots (CQDs)

To produce CQDs, lemon juice was obtained as the main carbon source. Firstly, 80 mL of extracted pulp-free lemon juice was added into a PTFE Teflon-lined autoclave containing 40 mL ethanol. To commence the hydrothermal treatment, the autoclave was placed into an oven and heated for three hours, at a temperature of 120 °C [18]. A dark brown suspension was formed after the hydrothermal treatment. An excessive amount of dichloromethane was added into the suspension and it was centrifuged to remove the unreacted organic moieties [19]. The addition of dichloromethane and the centrifugation process were repeated three times to wholly remove the organic moieties from the product solution. Lastly, a filter paper was used to remove the large particles from the diluted CQD solution.

### 2.3. Synthesis of Ag/CQDs Nanohybrids and Ag Nanoparticles 

To synthesize the Ag/CQDs nanohybrids, the prepared CQDs solution was added into a conical flask containing 150 mL of 0.02 M sodium borohydride (NaBH_4_). This was followed by dropping 50 mL of 0.01 M silver nitrate (AgNO_3_) solution into the conical flask with continuous stirring in an ice bath. The Ag/CQDs nanohybrids formed were rinsed with distilled water for four times and centrifuged prior drying in an oven. Similarly, the pure Ag nanoparticles were produced by applying the same method as the previous procedures. The only difference in synthesizing pure Ag nanoparticles was that the addition of CQDs solution into the conical flask was excluded in this process. The synthesized pure Ag nanoparticles and Ag/CQDs nanohybrids in solution and powder form are shown in Figure S1. The color of the Ag nanoparticle changed from silver to brown with the attachment of CQDs. 

### 2.4. Characterization of Sample Powder

After obtaining the dried sample powders, which were the Ag/CQD nanohybrids and pure Ag nanoparticles, they were characterized by means of several techniques. The structure or crystallinity of the nanomaterials was determined using X-ray diffraction analysis (XRD, XRD-6000, Shidmazu, Kyoto, Japan). The functional groups were identified by the means of Fourier transform infrared spectroscopy (FTIR, IS10, Nicolet, Madison, WI, USA). Scanning electron microscopy with energy dispersive X-ray spectroscopy (SEM-EDX, S-3400N, Hitachi, Tokyo, Japan) were employed to investigate the morphology and the presence of elements of the nanomaterials. In addition, the surface charge was measured with zeta potential analysis (Malvern, Nano ZS, Worcestershire, UK). The size of the CQDs are shown in Figure S2 with the use of transmission electron microscopy (TEM; Zeiss, Libra-120, Oberkochen, Germany).

### 2.5. Fabrication of Composite Membrane

A few sets of membranes were fabricated with different loadings of Ag/CQDs powder (0.3 wt %, 0.5 wt %, 0.7 wt %, and 1.0 wt %) as shown in Table 1. The amount of PSF polymer was fixed at 17 wt %, whereas the amount of PVP additive was maintained at 1 wt % with respect to the amount of PSF. 

The NIPS method was employed to synthesize the mixed matrix membrane. NMP, which acted as a solvent, was prepared in a conical flask. Then, the respective amounts of PVP and Ag/CQDs powders were sonicated prior to being added into the solvent and stirred to homogenize the mixture. After that, PSF granules were slowly added into the conical flask and further stirred at the temperature of 60 °C for four hours, followed by room temperature for 24 h [20]. After degassing for 24 h, the casting solution was spread uniformly on a clean glass plate and casted using a casting knife. The thickness of the polymer between the knife and the glass plate was maintained at about 0.2 mm. Subsequently, the glass plate, together with the casted polymer, was immersed immediately into a water bath, which acted as the nonsolvent coagulation bath. A transparent casted film was turned swiftly into a white-coloured membrane. Finally, the fabricated membrane was kept in a distilled water bath for further use [20]. The pictures of the membranes are shown in Figure S3. 

### 2.6. Characterization of Membrane

After completing the fabrication of the composite membrane, it was characterized by using several techniques, such as SEM analysis in conjunction with EDX, water contact angle analysis, and computation of porosity and pore size. Hydrophilicity is one of the critical parameters in evaluating the performance of the membrane. To identify the hydrophilicity of the membrane, water contact angles were measured by employing the sessile drop method [21]. The contact angle value, which is the angle between the water droplet and the membrane surface, was calculated by a video contact angle system, which used the DSA100 software (FM40Mk2, KRÜSS GmbH, Hamburg, Germany). 

To compute the overall membrane porosity, the gravimetric method was applied. The wet membrane was weighed, and the value was denoted as $W_{1}$. The wet membrane was dried at room temperature for one day. The dried membrane was weighed again, and the value was denoted as $W_{2}$. Furthermore, the thickness of the membrane, *l* was measured by using a micro thickness gauge. Finally, the overall membrane porosity, ε, was calculated by using Equation (1).

(1) $$\varepsilon =\frac{W_{1}-W_{2}}{A_{small}l\rho_{w}}$$

where $\rho_{w}$ is the density of distilled water, which is 998 kg/m^3^, and A is the active area of the membrane.

After that, the membrane pore size, *r_m_* was computed by using Equation (2) [22].

(2) $$r_{m}=\sqrt{\frac{\left(2.9-1.75\;\varepsilon \right)\times 8\;n\;l\;Q}{\varepsilon \times A\times \Delta P}}$$

where n is the viscosity of pure water in kg m/s, Q is the volume of the collected permeate water per unit time in m^3^/s, and ΔP is the transmembrane pressure in Pa.

### 2.7. Performance of Composite Membrane

Several experimental works and calculations were done to evaluate the performance of the membrane such as pure water flux, rejection test and membrane fouling analysis. To test the permeability of water across the fabricated membrane, a dead-end cell was used [21]. The water flux was then computed by using Equation (3) [21].

(3) $$J_{w}=\frac{Q}{A\Delta t}$$

where *J* is the permeate water flux ($L/m^{2}\;h$), *A* is the effective area of the testing membrane, which is 0.00146 $m^{2}$, and *t* is the time interval (h). Lastly, the permeability of the membrane, *P* ($L/m^{2}\;h\;\mathrm{bar}$), was computed by dividing the flux by the operating pressure. The pure water flux was conducted three times and the standard deviation was obtained.

To evaluate the membrane selectivity, a performance on the solute rejection of membrane was performed in the dead-end cell using 10 mg/L of tartrazine dye as the feed solution at 2 bar. The concentration of dye contained in the collected permeate solution was determined by using a UV-vis spectrophotometer [20]. The wavelength region of the tartrazine dye was in the range of 400 nm to 450 nm [23]. The solute rejection was calculated by using Equation (4) [21]. The rejection test was carried out three times and the standard deviation was obtained.

(4) $$R\left(\%\right)=\left(1-\frac{C_{p}}{C_{f}}\right)\times 100\%$$

where *R* is the rejection of solute (%), $C_{p}$ is the concentration of solute in the permeate solution (mg/L) and $C_{f}$ is the concentration of solute in the feed solution (mg/L). 

### 2.8. Membrane Fouling Analysis

To evaluate the antifouling ability of the membrane, fouling analysis was carried. Similar to the rejection test, 10 mg/L of tartrazine dye was used as the feed solution and operated at 2 bar. Initially, the filtration was run with distilled water and the value of the pure water flux was denoted as $J_{w1}$. Subsequently, the feed solution was replaced by tartrazine dye and the filtration process was repeated. This dye filtration process was carried out for three cycles. The weight of permeate was recorded every 30 s and the flux from the permeated dye was computed and denoted as $J_{dye}$. After that, the fouled membrane was cleaned using 1000 ppm of sodium hypochlorite solution (NaOCl) after completing each cycle. 

Lastly, the filtration process was run again by using distilled water to get the final value of pure water flux, $J_{w2}$. Eventually, the total flux decline rate (FDR), known as the total fouling, reversible fouling (RF) and irreversible fouling (IF), were computed by using Equations (5)–(7), respectively [14,24].

(5) $$\mathrm{FDR}=\left(1-\frac{J_{dye}}{J_{w1}}\right)\times 100\%$$

(6) $$\mathrm{RF}=\left(\frac{J_{w2}-J_{dye}}{J_{w1}}\right)\times 100\%$$

(7) $$\mathrm{IF}=\left(\frac{J_{w1}-J_{w2}}{J_{w1}}\right)\times 100\%$$

The membrane fouling analysis was implemented three times and the standard deviation was obtained.

## 3. Results and Discussion

### 3.1. Characterization of Sample Powder

#### 3.1.1. Structure and Crystallinity Analysis

**Figure 1:**
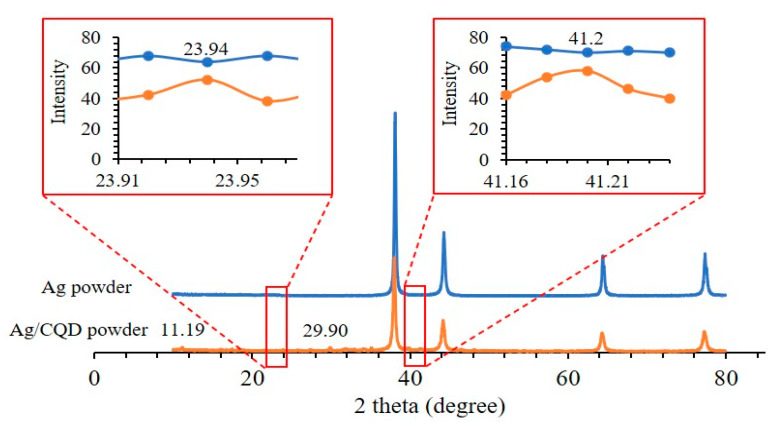
XRD pattern of Ag powder and Ag/CQD powder.

The XRD patterns of Ag and Ag/CQD powders are shown in Figure 1. Both Ag and Ag/CQD powders consisted of four obvious and narrow strong peaks that indicated the presence of Ag in the sample powder. The peaks located at 38.16°, 44.30°, 64.46° and 77.41° were ascribed to the (111), (200), (220) and (311) crystallographic planes of face-centered cubic of Ag nanoparticles, respectively [25]. With reference to the study by Mahmoudi, et al. (2019), the four peaks as depicted in the XRD spectra proved the structure of the Ag powder to be face-centered cubic (fcc). Therefore, the XRD graph clearly concluded that the Ag nanoparticles, which were generated by the reaction between sodium borohydride and silver nitrate solution, were in the crystalline phase in nature. Apart from the four obvious peaks that indicated the Ag crystalline phases, no other obvious peaks were found from the blue line graph in the XRD profile. Thus, no impurities were detected in the XRD graph [26].

The diffraction angle shifted from 38.16° to 38.04° after CQD is incorporated into the Ag. This indicated successfully intercalated CQD into the Ag. However, significant changes were not observed in the host structure due to the small amount of CQD. The results were in accordance with the study by Sim et al. (2018) [12] where the changes could hardly be noticed. The small and broad peaks located at 23.94° and 41.20° as shown in the inset were related to (002) and (101) diffraction patterns of graphitic carbon, respectively, in the Ag/CQD powder, which exhibited the amorphous nature of the CQDs. The diffraction patterns of the graphitic carbon were in accordance with the study by Tadesse et al. [19]. Furthermore, the small peak at 11.19° was assigned to the feature of the graphitic carbon and it was in close agreement with the report by Rajeswari et al. [27]. 

Apart from the presence of carbon and silver, there were some impurities existing in the Ag/CQD sample. Based on the study by Li et al. [21], the small peak at 29.90° could be related to the feature peak (104) of sodium nitrate, which was one of the by-products during the formation of Ag/CQD. The hydroxyl groups of the sodium nitrate solution might bond with the functional groups that had been attached on the CQDs. These components could not be removed completely despite several washes having been performed due to the strong hydrogen bonding between the functional groups of CQDs and the hydroxyl groups from the sodium compounds.

#### 3.1.2. Functional Group Analysis

**Figure 2:**
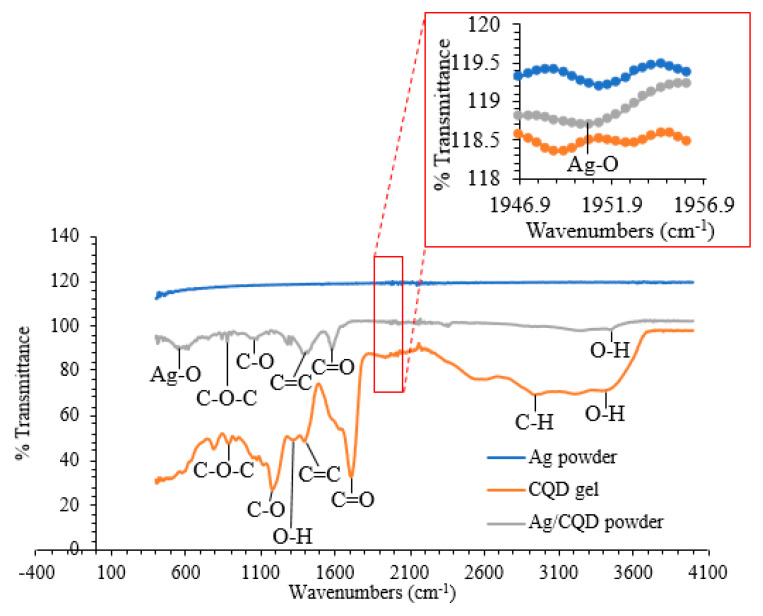
FTIR spectra of Ag and Ag/CQD powders.

The FTIR spectra of the Ag nanoparticles, CQD gel and Ag/CQD powder are depicted in Figure 2. The Ag nanoparticles did not absorb infrared light due to their lower net change in the bond’s dipole moment and they had the wavenumbers that were lower than the lower boundary of the mid-infrared ray used by the conventional FTIR equipment [28]. However, silver oxide, AgO was noticed in the inset of Figure 2, which verified the existence of the Ag nanoparticles. Both Ag and Ag/CQD spectra displayed the similar small and broad peak located at the wavenumber of about 1951 cm^−1^, which was associated with the Ag bonding with the oxygen molecules from the hydroxyl groups of CQDs [26]. Apart from that, the small peak located at 550.12 cm^−1^ could be related to the lattice vibration of AgO in the Ag/CQD spectra and it was in close agreement with the study by Siddiqui et al. [29].

For the spectra of CQD as shown in Figure 2, the small peak located at about 890 cm^−1^ was ascribed to the stretching vibration of the oxirane C-O-C band [30]. The peak at the spectrum of Ag/CQD powder diminished after being grafted with Ag nanoparticles [26]. The CQD spectrum indicated a strong peak at the wavenumber of 1169.66 cm^−1^, which originated from the stretching vibration of C-O functional groups [19]. The wavenumber shifted to 1076 cm^−1^ in Ag/CQD spectrum due to the formation of a chemisorption bond between C-O and the Ag nanoparticles that changed the electron distribution over the molecular orbitals [31]. Thus, the peak of the C-O functional group was weakened and shifted to the left.

Besides, the small and broad peak located at the 1302 cm^−1^ was seen in both Ag/CQD and CQD spectra. This peak could be attributed to the bending vibration of hydroxyl groups [25,30]. Besides, the CQD spectra illustrated a small peak at around 1414 cm^−1^ that could be assigned to the C=C stretching vibration. The presence of the C=C bond verified the existence of aromatic skeleton in the CQDs [19]. Generally, in the process of hydrothermal treatment, the pulp-free lemon juice was heated for the carbonization and dehydration process so that the carbon molecules gathered and bonded to each other to generate the basic aromatic skeleton of C=C or C–C bond. Then, the remaining molecules approached the surface of the nucleus to generate new C=C or C–C bonds continuously, to expand the structure of the basic framework until a complete structure of CQD was formed [18].

A very obvious and strong peak was located at the wavenumber of 1700 cm^−1^ in the CQD spectrum, which represented the existence of the carboxylic C=O stretching vibration [30]. On the other hand, in the Ag/CQD spectrum, the C=O bond originated at the peak of around 1600 cm^−1^. The diminution of the peak was observed in the Ag/CQD spectrum and the peak was shifted to the left when the CQD was successfully bonded with the Ag nanoparticles [32]. Lastly, the peaks located at 2964 cm^−1^ and 3450 cm^−1^ exhibited in the CQD spectrum corresponded to the stretching vibrations of aliphatic C–H bond and carboxylic O–H bond, respectively [18,19]. These two peaks started to diminish and disappear when the Ag/CQD was produced.

#### 3.1.3. Morphology and Elemental Analysis

**Figure 3:**
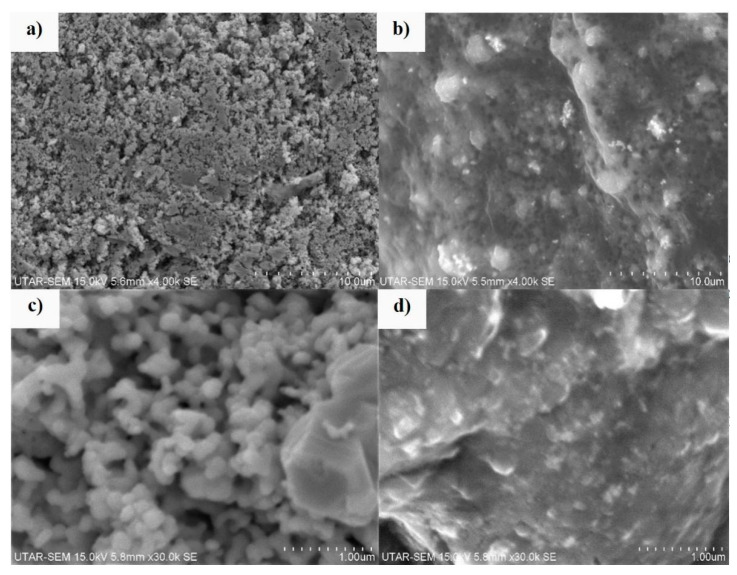
SEM images of (**a**) Ag powder and (**b**) Ag/CQD powder at the magnification of ×4 k, and (**c**) Ag powder and (**d**) Ag/CQD powder at the magnification of ×30 k.

Figure 3 illustrates the SEM images of the Ag powder and Ag/CQD powder at the magnification of ×30 k. As shown in Figure 3a, the Ag nanoparticles were powdery, and they were made up of discrete spherical particles. Referring to Figure 3c, with the absence of CQDs, the pure Ag, composed of smooth, spherical-shaped particles, was similar to the study reported by Yu, et al. [33]. 

Unlike the powdery Ag particles, Figure 3b indicates successful grafting of the CQD layer on the crystalline Ag nanoparticle as a layer of CQD on the surface of Ag was observed. The crystalline Ag nanoparticles were seen to be intermixed well with the CQD nanoparticles [34]. The CQD nanoparticles with a size smaller than 10 nm are presented in Figure S2. 

#### 3.1.4. Surface Charge Analysis

Zeta potential analysis was conducted to identify the surface charge of the powder. The surface charge of the powder played an important role in rejection of foulants in terms of the steric repulsion mechanism [22]. The average zeta potential of pure Ag, CQD and Ag/CQD nanoparticles are −13.13 ± 0.68 mV, 1.09 ± 0.17 mV and −33.43 ± 0.62 mV, respectively. Figure S4 show some of the zeta potential results of the synthesized nanomaterials. 

The pure Ag nanoparticles possessed negative zeta potential, which was mainly owing to the sodium borohydride that acted as a strong reducing agent in producing the Ag powder [35]. The value was in close agreement with the study by Abbaszadegan et al. [35]. According to a study by Gai, et al. [13], the zeta potential value of pure CQD nanoparticles should be negative. The CQD solution in this study experienced a very small net positive charge of 1.09 mV, which was near to zero charge (neutral charge). This was due to the existence of a substantial amount of water molecules, which experienced neutral charge, affecting the charge of the CQD nanoparticles. The water dissociated into ions and reacted with the function groups of CQD, and thus more positive ions were produced. When the positive zeta potential from the positive ions was dominant over the electronegativity of the negatively charged functional groups, such as the hydroxyl group and the carboxyl group, a net positive charge was generated [36].

Ag/CQD powder depicted the highest negative charge as compared to pure Ag and CQD nanoparticles. The negatively charged functional groups attached on the CQD, such as the carboxyl group (COO-) and the hydroxyl group (OH-), increased the electronegativity of the Ag/CQD particles. In addition, the negatively charged Ag nanoparticles further increased the electronegativity of the Ag/CQD nanohybrids [36]. Since the Ag/CQD nanohybrids were dried into powder form, the water content inside the sample was minimal and hence did not affect the surface charge of the sample.

The previous studies showed that nanocomposite with higher negative charge and greater hydrophilic functional groups would improve the membrane wettability, permeability, and rejection ability [8,24,37]. As the Ag/CQDs powder showed higher negative charge compared to the pure Ag nanoparticles, hence the membrane fabrication and characterization will only focus on the composite powder in this study.

### 3.2. Characterization of Membranes

#### 3.2.1. Water Contact Angle, Pore Size and Porosity

Table 2 shows the water contact angle, pore size and porosity of the membranes. Basically, the water contact angle was measured to evaluate the hydrophilicity of each membrane. Generally, the higher the affinity of the membrane towards the water, the lower the water contact angle [38].

The pure PSF membrane indicated a higher water contact angle of 80.6° ± 2.15°, which was very near to 90°. The PSF polymer was hydrophobic, and little amount of hydrophilic PVP in the membrane was not adequate to improve the hydrophilicity of the membrane [39]. The modified membrane with 0.5 wt % of Ag/CQD showed the lowest water contact angle of 67.4°, indicating the most hydrophilic membrane among the five membranes. Overall, the existence of hydrophilic Ag/CQD additives had reduced the contact angle of all the modified membranes and improved their hydrophilicity. The effect of the addition of Ag/CQD on hydrophilicity of the membrane was obvious when the membrane with 1.0 wt % of Ag/CQD showed a lower contact angle compared to the pure PSF, although its pore size is smaller. Basically, the functional groups of the CQDs on the membrane surface and pore walls would attract the water molecules by forming strong hydrogen bonds with them and pulling them towards the membrane matrix [40,41]. Apart from CQDs, liberated silver ions are also capable of enhancing the hydrophilicity of the membrane [42].

As presented in Table 2, when the loading of the Ag/CQD powder increased, the pore size of the membrane increased. This implied that higher loading of Ag/CQD additives could enhance the mass transfer between the solvent and non-solvent and hence enlarge the pore size of the membrane. However, as the loading of Ag/CQD powder reached beyond 0.5 wt %, the pore size started to decrease. This was attributed to the higher amount of Ag/CQD powder loading, increasing the viscosity of the membrane casting solution. The higher viscosity of the solution delayed the mass transfer between the solvent and non-solvent, hence it hindered the large pore formation during the phase inversion process [8].

#### 3.2.2. Functional Group Analysis of Membranes

**Figure 4:**
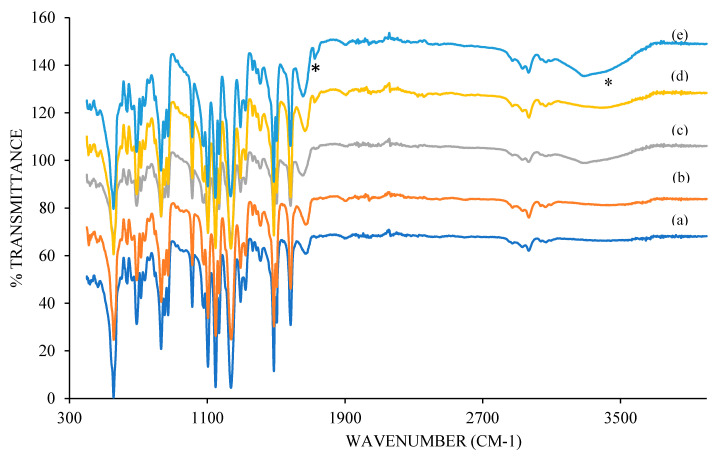
FTIR spectrum of membranes with (**a**) 0 wt % (PSF); (**b**) 0.3 wt %; (**c**) 0.5 wt %; (**d**) 0.7 wt %; (**e**) 1.0 wt % of Ag/CQD. ∗ (left) 1700 cm−1, C=O stretching vibrations; ∗ (right) 3430 cm−1, O–H stretching vibrations.

Figure 4 shows the FTIR spectrum of different loadings of Ag/CQD membranes in the wavelength range of 400–3900 cm^−1^. All the pure PSF signature peaks are visible in the composite membranes [43]. The peak at 1585 cm^−1^ indicates the characteristic stretch of aromatic ring quadrant. The peak of 1247 cm^−1^ is associate with phase stretching of sulfone (SO_2_), while 1013 cm^−1^ represents C=O=C aryl out of phase stretch. The peak at 834 cm^−1^ means aromatic para-substituted adjacent H wagging. The peak of 690 cm^−1^ and 558 cm^−1^ is associated with C–S–C out of phase stretch and SO_2_ scissors deformation, repectively. This showed that the embedment of the nanomaterial did not disrupt the polymer structure. Besides, FTIR spectra of the composite membrane reveals a broad peak at 3430 cm^−1^ and sharper peak around 1700 cm^−1^, which corresponds to O–H and C=O stretching vibrations, respectively [44]. The existence of these peaks was due to the presence of Ag/CQD powder as discussed in Section 3.1.2. Thus, it could be confirmed that the incorporation of the Ag/CQD powder had improved the hydrophilicity of the membrane. 

#### 3.2.3. Structure and Elemental Analysis of Membranes

**Figure 5:**
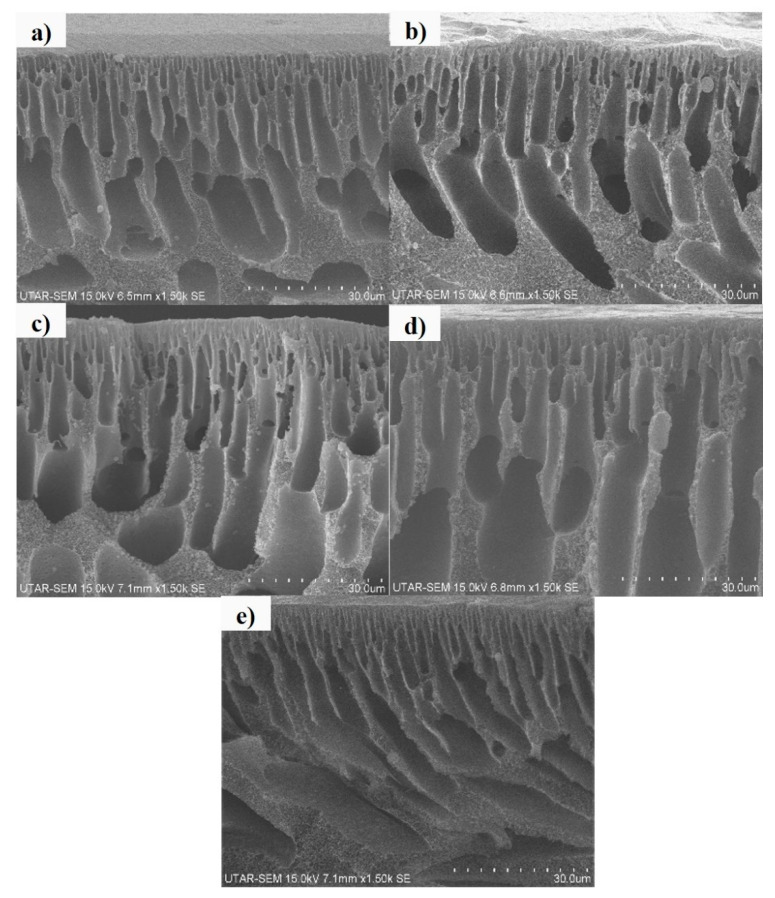
Cross-sectional view of membranes: (**a**) 0 wt % (PSF); (**b**) 0.3 wt %; (**c**) 0.5 wt %; (**d**) 0.7 wt %; (**e**) 1.0 wt % of Ag/CQD at the magnification of ×1.50 k.

Figure 5 illustrates the cross-sectional view of the membranes with different loadings of Ag/CQD powder. The cross-sectional view of the membranes indicated that the membranes were composed of two layers, which were the dense top layer and porous sublayer. The sublayer consisted of finger-like cavities and macrovoids which formed due to the instantaneous demixing process, as there was high mutual affinity between the NMP solvent and water coagulation bath [20,45].

Pure PSF membrane was made up of a dense skin layer, as shown in Figure 5a. Without the presence of Ag/CQD, the pores of the membrane were very small and highly dense due to the slow exchange of NMP and water bath during the immersion process, leading to the aggregation of polymer at the top layer [46]. On the other hand, with an appropriate concentration of Ag/CQD, the skin layer was less dense, and the pores, macrovoids and cavities were enlarged during the immersion process.

The membrane with 0.5 wt % of Ag/CQD (Figure 5c) shows wide and deep finger-like cavities as compared to other membranes. This observation was in an agreement with the membrane pore size, as discussed in Section 3.2.1. When the loading of Ag/CQD increased beyond 0.5 wt %, the pore size at the top layer of the membrane reduced, as shown in Figure 5d,e.

**Figure 6:**
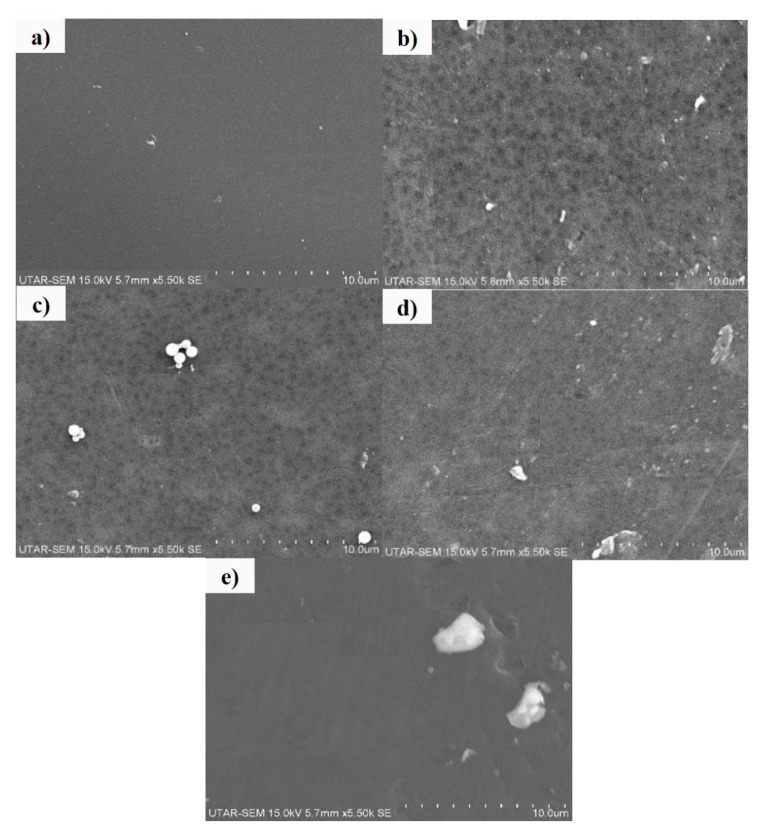
Top view of membranes: (**a**) 0 wt % (PSF); (**b**) 0.3 wt %; (**c**) 0.5 wt %; (**d**) 0.7 wt %; (**e**) 1.0 wt % of Ag/CQD at the magnification of ×5.50 k.

Figure 6 demonstrates the top view of the membranes with different loadings of Ag/CQD powder at the magnification of ×5.50 k. As shown in Figure 6a,e, the pore of the pure PSF membrane and the membrane with 1.0 wt % of Ag/CQD were too small to be seen in the SEM images. For the rest of the membranes, the SEM images presented a well and uniform distribution of pores in the membranes due to rapid mass transfer happening during the phase inversion process [47]. Apart from that, bigger pore size was observed for membranes with 0.3 wt %, 0.5 wt % and 0.7 wt %. The observation was in accordance with the membrane pore size shown in Table 3. 

The elemental analysis of the composite membrane with 0.5 wt % of Ag/CQD powder is shown in Figure S5. The composite membrane was mainly composed of carbon and oxygen elements, which were 76.3 wt % and 17.62 wt %, respectively. Both elements were mainly originated from PSF polymer and CQD nanoparticles. The sulphur element (5.73 wt %) was mainly derived from the PSF compound. Apart from that, silver elements 0.35 wt % was detected, indicating the presence of Ag in the membrane.

**Figure 7:**
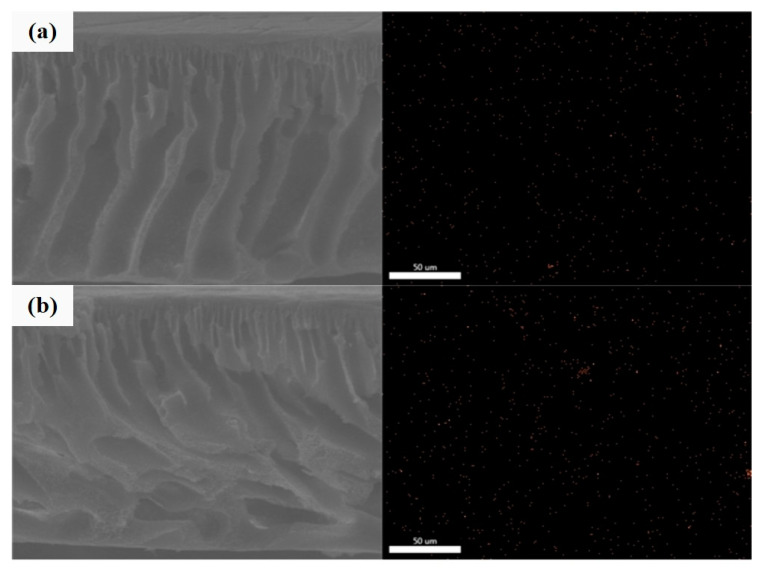
EDX mapping of the Ag element in the membranes with (**a**) 0.5 wt % and (**b**) 1.0 wt % loadings.

EDX mapping was performed to identify the distribution of the Ag element in the membranes, as depicted in Figure 7. The composite membranes’ sectional view shows higher density of Ag element in the membrane with 1.0 wt % Ag/CQD loading. It was noticed that the Ag element is distributed uniformly over both membranes. 

### 3.3. Performances of the Membranes

#### 3.3.1. Membrane Permeability 

**Figure 8:**
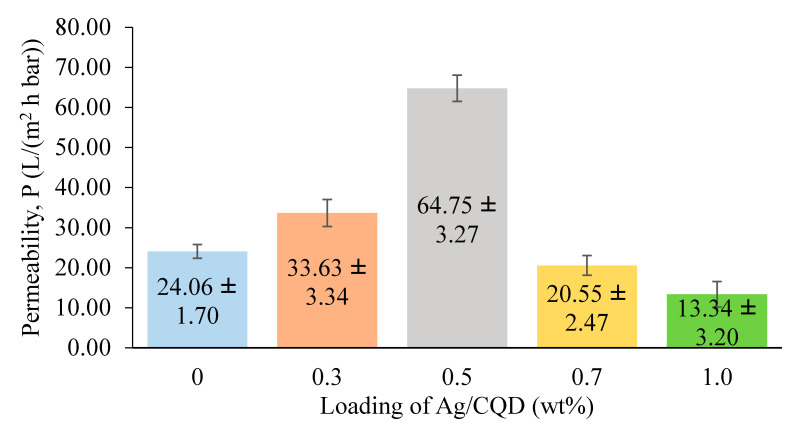
Permeability of the membranes with different loadings of Ag/CQD.

The permeabilities of the membranes with different loadings of Ag/CQD powders are displayed in Figure 8. Overall, the permeability of the membranes increased with the loading of Ag/CQD and decreased beyond the optimum point. The permeability of the membrane with 0.5 wt % of Ag/CQD powder was found to be the highest, with 169% improvement. The hydrogen bonding formation between the functional groups of CQDs and the water molecules strengthened the driving force for water transport in the water channels to enhance the water permeability [36]. This verified that the incorporation of Ag/CQD indeed improved the hydrophilicity of the membrane, which enhanced the adhesion and absorption of water into the membrane matrix [41]. Apart from that, this membrane also exhibited the biggest membrane pore size. This characteristic could further enhance the permeability of the membrane as higher volumes of water could pass through the membrane [40].

On the other hand, the membrane with 0.3 wt % of Ag/CQD possessed the second highest permeability due to its relatively large pore size. Although it had a higher water contact angle than the membrane with 1.0 wt % of Ag/CQD, the difference of the water contact angle between them was not huge enough to see the difference of their permeabilities. As a comparison, the pore size of the membrane became a more dominant parameter.

On the other hand, the membrane with 1.0 wt % of Ag/CQD had the least permeability, which was around 13.34 L/(m^2^ h bar). This might due to the relatively small pore size, making the water difficult to diffuse into the pore length of the membrane [41]. When the loading of Ag/CQD was beyond 0.5 wt %, the permeability decreased due to the higher polymer solution viscosity, resulting in smaller pore size. As for the pure PSF membrane, although it had the highest water contact angle, due to its considerably large pore size, its permeability was higher than the membrane with 1.0 wt % of Ag/CQD loading. In conclusion, the permeability of the membranes was greatly affected by the loading of additives, pore size and water contact angle of the membranes, depending on which parameter was more dominant than the other one.

Table 3 presents previous studies that modified the PSF membrane by blending with different nanomaterials. Generally, the incorporation of these carbon-based nanomaterials has improved the hydrophilicity of the pristine PSF polymer and hence improved the permeability.

**Table 3:** Comparison of permeability improvement of various modified PSF membranes.

Membrane	Permeability (Lm^−2^ h^−1^ bar^−1^)	Improvement (%)	Reference
PSF PSF + 2 wt % multi-walled carbon nanotube (MWCNT)	~71 ~149	+108	[48]
PSF PSF + 5 wt % MWCNT	~5.5 ~12.5	+127	[49]
PSF PSF + silver/graphene oxide (Ag/GO)	~16 ~28	+75	[8]
PSF PSF + 1.5 w/wt % sulfonated GO	78 175.2	+125	[50]
PSF PSF + 0.3 wt % GO QD	130.54 82.52	+60	[17]
PSF PSF + 0.5 wt % Ag/CQD	24.06 64.75	+169	This study

#### 3.3.2. Membrane Rejection Test

**Figure 9:**
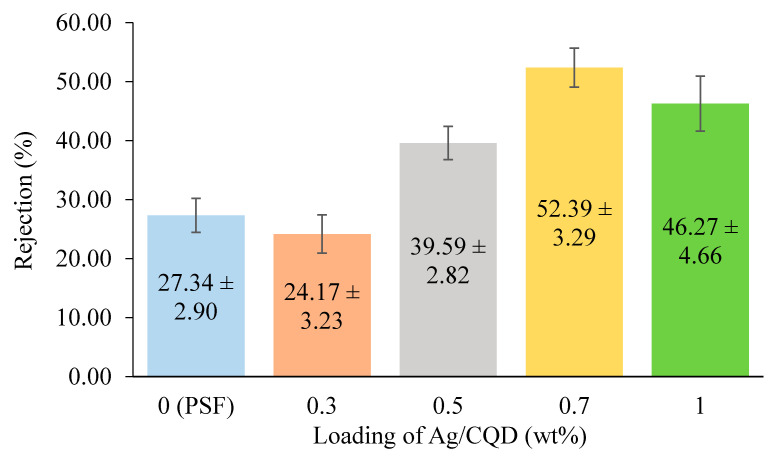
Tartrazine dye rejections of the membranes with different loadings of Ag/CQD.

Rejection test was conducted to evaluate the ability of the membranes in removing tartrazine dye. Tartrazine is an anionic dye [51] and therefore a negatively-charged membrane is required to remove this dye efficiently. Referring to Figure 9, the membrane with 0.7 wt % of Ag/CQD had the highest rejection of tartrazine dye, followed by the membranes with 1.0 wt %, 0.5 wt %, 0 wt % and lastly 0.3 wt % of Ag/CQD. Coupled with the moderately high water contact angle, the membranes with 0.7 wt % and 1.0 wt % of Ag/CQD exhibited better tartrazine rejections as compared to others. The highest rejection improved 92% using 0.7 wt % of Ag/CQD membrane compared with the pure PSF membrane. The improvement was huge compared with some previous studies. According to the study by Sharma and Purkait (2016) [52], the permeability of membrane incorporated with dextro-tartaric acid increased by double. However, the dye rejection was only increased by 11%. Another study by Fatima Anis et al. (2020) [53] reported that the increment of permeability and dye rejection using nano zeolite-Y-incorporated PSF membrane was 75% and 66%, respectively, as compared to the pure PSF membrane. This might be because the synthesized membranes (with 0.7 wt % and 1.0 wt % of Ag/CQD) had very small membrane pore sizes, causing the tartrazine to be very difficult to channel into the pore length of the membranes [40]. Besides, the electronegativity of the membrane increased with the loading of the negatively charged Ag/CQD [14]. 

As for the membrane with 0.3 wt % of Ag/CQD, it experienced the poorest tartrazine rejection of 24.17% ± 3.23%, as shown in Figure 9. The membrane pore size parameter was dominant over the surface charge as the concentration of additives was not sufficient to increase its electronegativity to reject the tartrazine particles from the membrane, due to the weak repulsion force [22]. In fact, the membrane pore size was larger for the tartrazine particles to channel through the pore length of the membrane. 

Although the membrane with 0.5 wt % of Ag/CQD was made up of large membrane pores, it showed a moderately high rejection as well. Coupled with the optimum amount of Ag/CQD loading, the negative zeta potential might be adequate for the formation of strong repulsion force between the membrane surface and the water molecules. Despite the absence of Ag/CQD, the pure PSF membrane experienced a rejection of 27.34%, which was slightly higher than the membrane with 0.3 wt % of Ag/CQD. This was due to the smaller membrane pore sizes that could avoid the tartrazine dye to pass through the membrane. 

In conclusion, the smaller the membrane pore sizes, the better the rejections of the tartrazine particles. Furthermore, the higher electronegativity of membrane could further improve the tartrazine rejection as it could strengthen the electrostatic repulsive force between the foulants and membrane surfaces [22].

#### 3.3.3. Fouling Analysis

**Figure 10:**
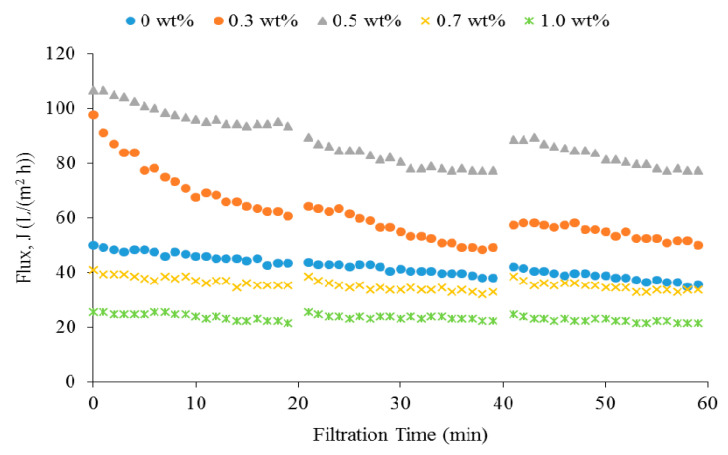
The flux of the membranes during the three cycles of dye filtration processes.

Membrane fouling analysis was carried out by using the anionic tartrazine dye as the feed solution. Figure 10 depicts the membrane flux for three cycles of dye filtration processes. The flux decline of pure PSF membrane and the membrane with 0.7 wt % and 1.0 wt % of Ag/CQD were less pronounced compared to the other membranes. They had very small flux decline due to their relatively small pore size where tartrazine particles were hard to penetrate the membrane pore [22,54]. The membranes could be easily recovered after rinsing with sodium hypochlorite solution as the cleaning agent. It is worth mentioning that the membranes with Ag/CQD showed a lower flux decline rate than the pure PSF membrane due to their negative charge, which enhanced the rejection of the tartrazine dye.

It can be observed that although the flux decline of the membranes with 0.3 wt % and 0.5 wt % of Ag/CQD is higher than the other membranes, the flux stayed high throughout the filtration process within the same period of time. The trend is in accordance with a study done by Chong et al. [55]. However, generally the membrane with bigger pore size would foul easily since the foulant particles could easily penetrate through the membrane and deposit on the pores surface [40,56]. Hence, the membrane flux decreased. Since the membrane with 0.5 wt % of Ag/CQD was composed of higher Ag/CQD loading, it possessed higher electronegativity and hence showed lower reduction in flux.

**Figure 11:**
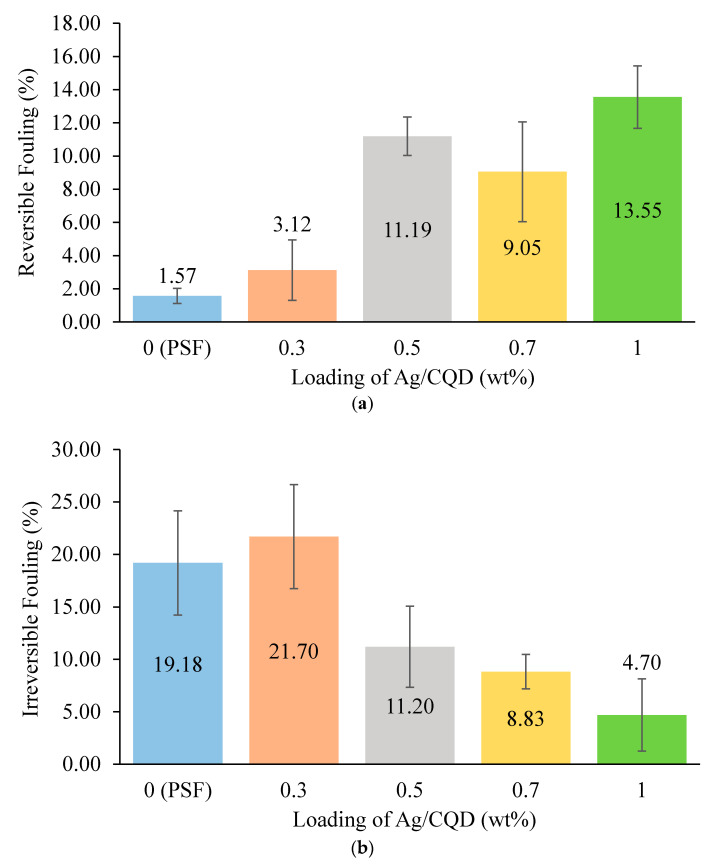
(**a**) Reversible and (**b**) irreversible fouling of the membranes with different loadings of Ag/CQD.

In order to quantitatively evaluate the antifouling performance of the membrane, the reversible fouling and irreversible fouling of the membranes were computed and presented in Figure 11. Membrane with no or low loading of Ag/CQD showed high irreversible fouling and high reversible fouling. As the electronegativity of these membranes was low, the tartrazine foulants firmly attached on the pore walls and were hardly removed during the cleaning process [22,54]. Besides, the pore size of the membrane with 0.3 wt % of Ag/CQD was larger and therefore was prone to irreversible fouling owing to the ease in accessibility of the foulants into the membrane [25,40].

On the contrary, the membranes with 0.5 wt %, 0.7 wt % and 1.0 wt % of Ag/CQD showed better reversible fouling and lower irreversible fouling. This was because they possessed higher electronegative charge and they could be easily recovered after the cleaning process [22]. However, as the membrane with 0.5 wt % of Ag/CQD was made up of larger membrane pore sizes, it experienced slightly high irreversible fouling. Therefore, it can be concluded that the size of the pores and the electronegativity of the membrane played vital roles in enhancing the antifouling ability of the membranes. 

## 4. Conclusions

This study has successfully synthesized CQD and Ag/CQD powder. The chemical and physical characteristics of the nanomaterials were determined. The SEM-EDX, XRD and FTIR analysis verified the presence of CQD attached to the Ag nanoparticle. Furthermore, the Ag/CQD powder showed high negative charge with the addition of CQD. PSF polymeric membranes with five different loadings of Ag/CQD were fabricated and well characterized with water contact angle analysis, pore size, FTIR and SEM-EDX analysis. All the Ag/CQD-modified membranes showed higher hydrophilicity than the pure PSF membrane. 

The permeability of the membranes was greatly influenced by the loading of additives, pore size and water contact angle of the membranes, depending on which parameter was more dominant than the other. Overall, the membrane with 0.5 wt % of Ag/CQD exhibited the lowest water contact angle with wide and deep finger-like cavities, therefore showing the highest permeability among the membranes. On the other hand, the rejection ability of the membrane was governed by the membrane pore size and surface charge. The modified membrane with smaller pore size and higher negative charge experienced higher rejection towards tartrazine dye. In the aspect of fouling analysis, the membrane with a higher loading of Ag/CQD experienced better reversible fouling and low irreversible fouling due to smaller membrane pore size or higher negative surface charge. 

This study showed that the incorporation of Ag/CQD into membrane could enhance the membrane’s permeability, rejection and anti-fouling ability. In future, CQD produced from biomass should be explored and developed as a more sustainable nanomaterial for membrane fabrication technology.

## Figures and Tables

**Table 1:** The loading of polysulfone (PSF), polyvinylpyrrolidone (PVP), N-methyl-2-pyrrolidone (NMP) and Ag/CQDs (silver/carbon quantum dots) nanohybrids.

Set	Ag/CQDs Nanohybrids (wt %)	PSF Polymer (wt %)	PVP Additive (wt %) (Respect to PSF)	NMP Solvent (wt %)
1	0	17	1	83.0
2	0.3	17	1	82.7
3	0.5	17	1	82.5
4	0.7	17	1	82.3
5	1.0	17	1	82.0

**Table 2:** Water contact angle, pore size and porosity of the membranes with different loadings of Ag/CQD powder.

Loading of Ag/CQD (wt %)	Water Contact Angle (°)	Pore Size, *r* (nm)	Porosity, *ε*
0 (Pure PSF)	80.6 ± 2.15	8.51	0.8965
0.3	73.9 ± 1.04	13.34	0.8782
0.5	67.4 ± 1.57	20.14	0.8239
0.7	74.0 ± 1.19	11.83	0.8227
1.0	72.0 ± 1.06	7.16	0.7237

## Supplementary Materials

The following are available online at https://www.mdpi.com/2077-0375/10/8/175/s1, Figure S1: (a) dark brown suspension after hydrothermal treatment (b) clean CQD solution after extraction of organic moieties, (c) Ag powder and (d) Ag/CQD powder; Figure S2: TEM image of CQD; Figure S3: Fabricated membranes with (a) 0 wt% (PSF); (b) 0.3 wt%; (c) 0.5 wt%; (d) 0.7 wt%; and (e) 1.0 wt% of Ag/CQD loading; Figure S4: Zeta potential of (a) Ag, (b) CQD and (c) Ag/CQD; Figure S5: The Elemental Composition of the Membrane with 0.5 wt% of Ag/CQD.
