# Supplementary material for: Novel Carbon Quantum Dots/Silver Blended Polysulfone Membrane with Improved Properties and Enhanced Performance in Tartrazine Dye Removal
Source: Membranes (Basel). 2020 Aug 3;10(8):175. doi: 10.3390/membranes10080175 (PMC7465473; doi:10.3390/membranes10080175)
Supplement: Supplementary file 1 [file membranes-10-00175-s001.pdf]

membranes

Supplementary Materials: Novel Carbon Quantum Dots/Silver Blended Polysulfone Membrane with Improved Properties and Enhanced Performance in Tartrazine Dye Removal

Jin Yee Gan [1], Woon Chan Chong [1,*], Lan Ching Sim [1], Chai Hoon Koo [2], Yean Ling Pang [1], Ebrahim Mahmoudi [3,4] and Abdul Wahab Mohammad [3,4]

[1] Department of Chemical Engineering, Lee Kong Chian Faculty of Engineering and Science, Universiti Tunku Abdul Rahman, Jalan Sungai Long, Bandar Sungai Long, Cheras, 43000 Kajang, Selangor, Malaysia; cannygan97@1utar.my (J.Y.G.); simcl@utar.edu.my (L.C.S.); pangyl@utar.edu.my (Y.L.P.)

[2] Department of Civil Engineering, Lee Kong Chian Faculty of Engineering and Science, Universiti Tunku Abdul Rahman, Jalan Sungai Long, Bandar Sungai Long, Cheras, 43000 Kajang, Selangor, Malaysia; kooch@utar.edu.my

[3] Chemical Engineering Programme, Faculty of Engineering and Built Environment, Universiti Kebangsaan Malaysia, 43600 Bangi, Selangor, Malaysia; ebi.dream@gmail.com (E.M.); wahabm@eng.ukm.my (A.W.M.)

[4] Research Center for Sustainable Process Technology (CESPRO), Faculty of Engineering and Built Environment, Universiti Kebangsaan Malaysia, 43600 Bangi, Selangor, Malaysia

* Correspondence: chongwoonchan@gmail.com

Received: 7 May 2020; Accepted: 23 June 2020; Published: date

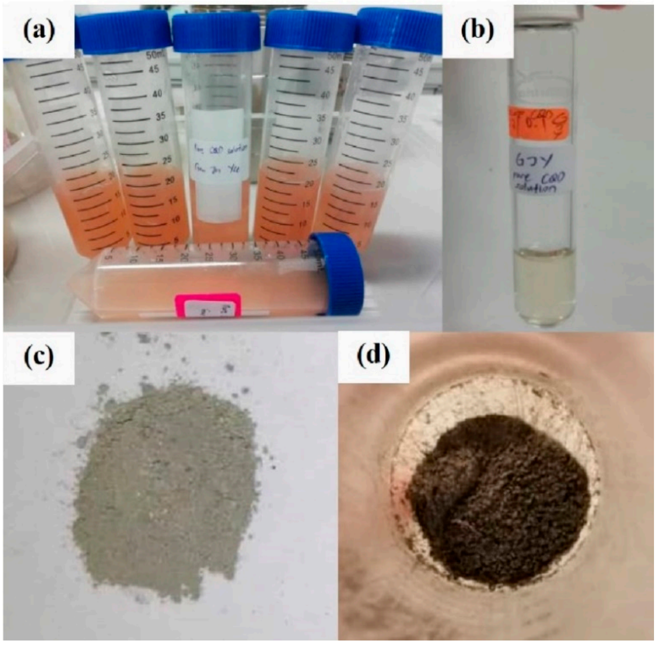

Figure S1. (a) dark brown suspension after hydrothermal treatment **(b)** clean CQD solution after extraction of organic moieties, **(c)** Ag powder and **(d)** Ag/CQD powder

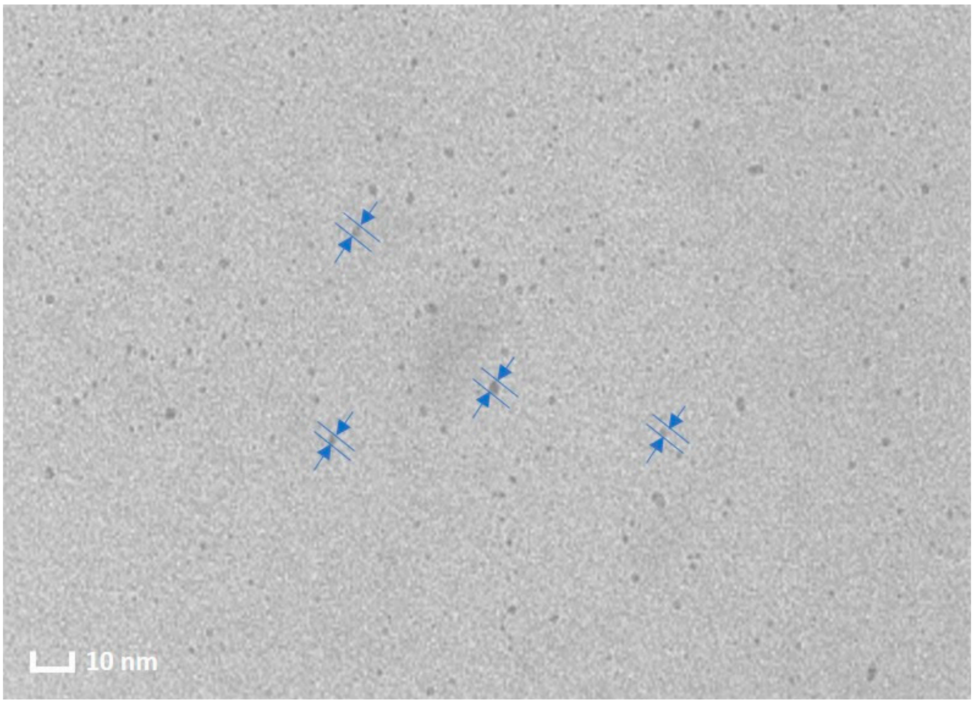

Figure S2. TEM image of CQD.

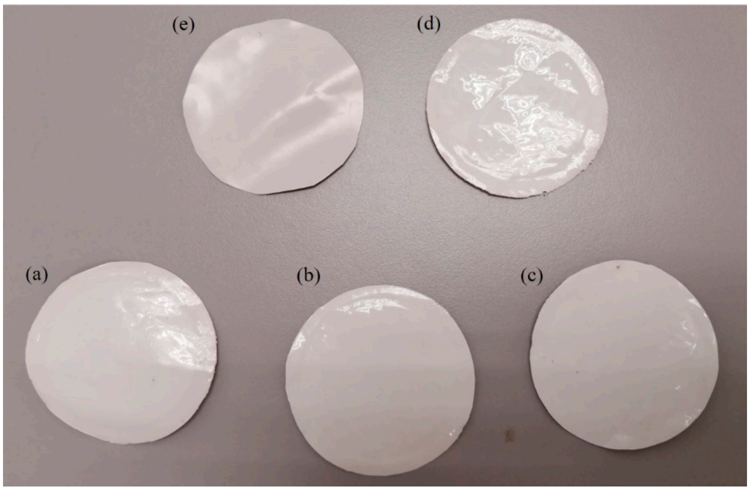

Figure S3. Fabricated membranes with (**a**) 0 wt% (PSF); (**b**) 0.3 wt%; (**c**) 0.5 wt%; (**d**) 0.7 wt%; and (**e**) 1.0 wt% of Ag/CQD loading.

membranes

(a)

Results

Zeta Potential (mV):	-14.1
Zeta Deviation (mV):	5.46
Conductivity (mS/cm):	0.0323
Result quality :	Good

	Mean (mV)	Area (%)	St Dev (mV)
Peak 1:	-14.1	100.0	5.46
Peak 2:	0.00	0.0	0.00
Peak 3:	0.00	0.0	0.00

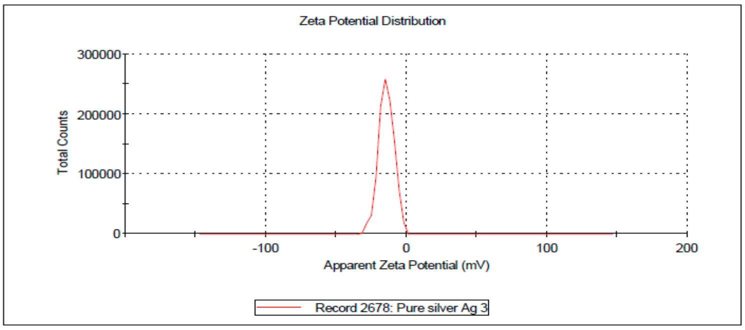

(b)

Results

Zeta Potential (mV):	1.25
Zeta Deviation (mV):	5.13
Conductivity (mS/cm):	3.05
Result quality :	Good

	Mean (mV)	Area (%)	St Dev (mV)
Peak 1:	1.25	100.0	5.13
Peak 2:	0.00	0.0	0.00
Peak 3:	0.00	0.0	0.00

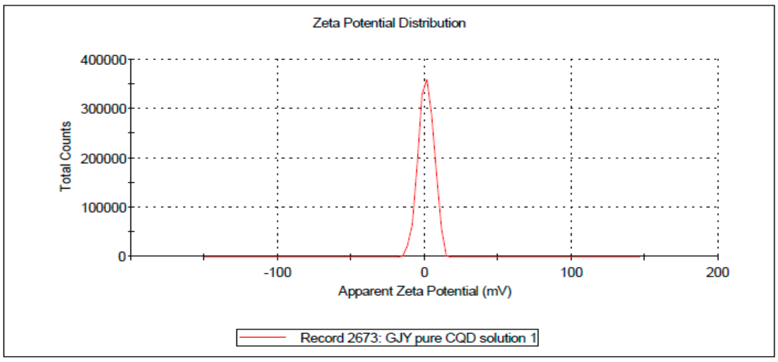

(c)

Results

Zeta Potential (mV):	-33.6
Zeta Deviation (mV):	5.71
Conductivity (mS/cm):	0.203
Result quality :	Good

	Mean (mV)	Area (%)	St Dev (mV)
Peak 1:	-33.6	100.0	5.71
Peak 2:	0.00	0.0	0.00
Peak 3:	0.00	0.0	0.00

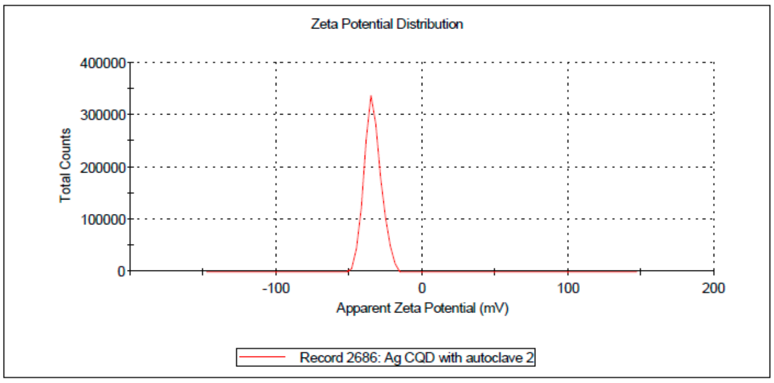

Figure S4. Zeta potential of (**a**) Ag, (**b**) CQD and (**c**) Ag/CQD

membranes

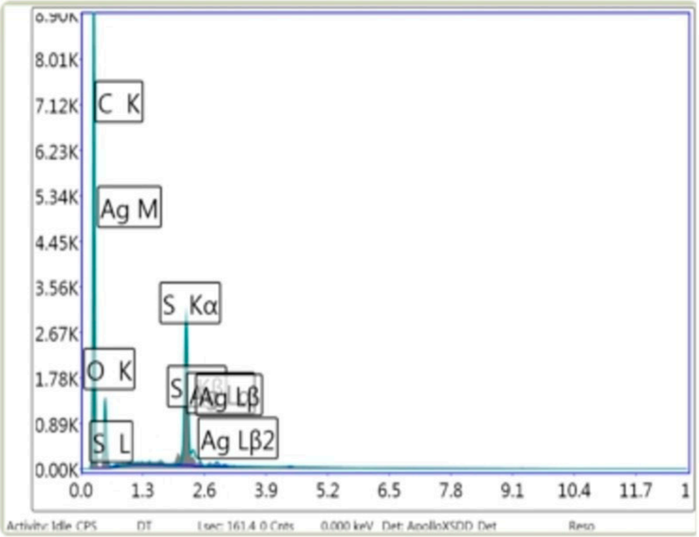

Element	Weight %	Atomic %
C K	76.3	83.2
O K	17.62	14.42
S K	5.73	2.34
AgL	0.35	0.04

Figure S5. The Elemental Composition of the Membrane with 0.5 wt% of Ag/CQD.
